# SARS-CoV-2 spike protein induces cognitive deficit and anxiety-like behavior in mouse via non-cell autonomous hippocampal neuronal death

**DOI:** 10.1038/s41598-022-09410-7

**Published:** 2022-03-31

**Authors:** Junyoung Oh, Woo-Hyun Cho, Ellane Barcelon, Kwang Hwan Kim, Jinpyo Hong, Sung Joong Lee

**Affiliations:** 1 Department of Neuroscience and Physiology, Dental Research Institute, School of Dentistry, Seoul National University, 1 Gwanak-ro, Gwanak-gu, Seoul, 08826 Republic of Korea; 2 Department of Brain and Cognitive Sciences, College of Natural Sciences and School of Dentistry, Seoul National University, Seoul, 08826 Republic of Korea; 3 OATC Research Center for Neurodiseases, Seoul, 08833 Republic of Korea

**Keywords:** Glial biology, Molecular neuroscience, Neuroimmunology

## Abstract

Severe acute respiratory syndrome coronavirus 2 (SARS-CoV-2) infection is accompanied by chronic neurological sequelae such as cognitive decline and mood disorder, but the underlying mechanisms have not yet been elucidated. We explored the possibility that the brain-infiltrating SARS-CoV-2 spike protein contributes to the development of neurological symptoms observed in COVID-19 patients in this study. Our behavioral study showed that administration of SARS-CoV-2 spike protein S1 subunit (S1 protein) to mouse hippocampus induced cognitive deficit and anxiety-like behavior in vivo. These neurological symptoms were accompanied by neuronal cell death in the dorsal and ventral hippocampus as well as glial cell activation. Interestingly, the S1 protein did not directly induce hippocampal cell death in vitro. Rather, it exerted neurotoxicity via glial cell activation, partially through interleukin-1β induction. In conclusion, our data suggest a novel pathogenic mechanism for the COVID-19-associated neurological symptoms that involves glia activation and non-cell autonomous hippocampal neuronal death by the brain-infiltrating S1 protein.

## Introduction

People worldwide are currently suffering from the coronavirus disease 2019 (COVID-19) pandemic caused by severe acute respiratory syndrome coronavirus 2 (SARS-CoV-2)^1,2^. Increasing evidence has shown that COVID-19 patients not only manifest respiratory-related symptoms, but also develop neurological and psychiatric symptoms, depending on the stage of infection, ranging from headache to cognitive and mood disorders^3,4^. According to clinical studies, 19% and 14% of COVID-19 patients develop depression and anxiety, respectively^5^ and 10–20% suffer from cognitive impairment^6^. Therefore, it is obvious that SARS-CoV-2 somehow affects the central nervous system (CNS), but the molecular and cellular mechanisms are still elusive. Previous studies suspected direct SARS-CoV-2 infection into the CNS, as SARS-CoV-2 spike protein and transcripts were detected in post-mortem brains. Then, as a port of CNS entry, SARS-CoV-2 invasion via olfactory receptor neurons was proposed^7^. However, a recent study using unbiased transcriptome analysis of the post-mortem brain tissue of COVID-19 patients did not succeed in detecting molecular traces of SARS-CoV-2 virus in the brain parenchyma^8^ negating direct SARS-CoV-2 infection into the CNS parenchyma. More recently, it was reported that intravenously administered radiolabeled S1 subunit of SARS-CoV-2 spike protein (S1 protein) can translocate into brain parenchyma by crossing the blood–brain barrier^9^. Therefore, this suggests the possibility that S1 proteins translocated into the brain parenchyma may affect brain functions, which might underlie the neurological or psychiatric symptoms of COVID-19 patients. The possibility was examined by introducing S1 proteins into mouse brains. We showed that the injection of S1 protein into mouse hippocampus induced cognitive deficits and anxiety-like behaviors. As mechanisms, we found that SARS-CoV-2 S1 protein exerted non-cell autonomous hippocampal neuronal cell death by inducing interleukin-1 beta (IL-1β) expression from glial cells.

## Results

### SARS-CoV-2 spike protein induces cognitive decline and anxiety-like behavior in mice

**Figure 1:**
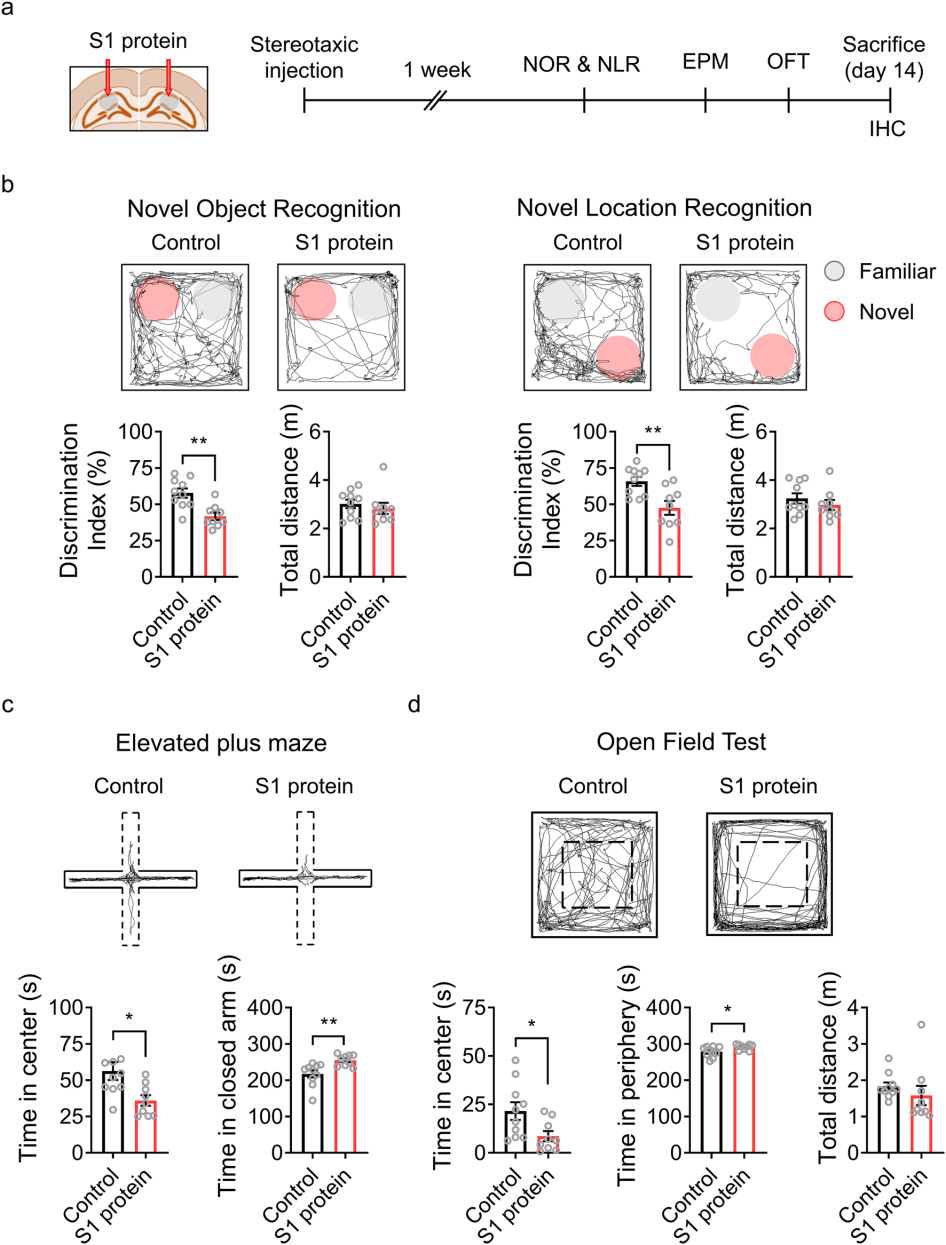
Administration of SARS-CoV-2 S1 protein induces cognitive deficit and anxiety-like behavior in mice. (**a**) Experimental design of S1 administration and behavioral tests. S1 protein (n = 10) or saline (Control, n = 10) was administered to C57BL/6 mice at 8 weeks of age, and behavioral tests were started 1 week after the administration. (**b**) Cognitive deficits were assessed using novel object recognition (NOR, left) or novel location recognition (NLR, right) tests and are presented as discrimination index percentages. Locomotor activity was measured by the total distance moved by mice in the chamber within a test session (bottom). The exploration time of a novel object or location divided by total exploration time was presented as the discrimination index of the novel object or location. The pink circle indicates a novel object or novel location. Anxiety-like behavior was assessed using the elevated plus maze test (EPM, panel **c**) and the open field test (OFT, panel **d**). (**c**) EPM trace of Control and S1 protein-injected groups (upper); the time spent in the open arm (dotted) vs. the closed arm (solid) in the EPM (bottom). (**d**) The center and periphery zones in the OFT are shown (upper). The time spent in the center vs. periphery zones in the OFT was examined (bottom). All representative navigations on the behavior test for a 5-min period are presented for the mice. The data are presented as the mean ± s.e.m. Statistical results are for unpaired *t*-tests. IHC, immunohistochemistry. ** *p* < 0.01, * *p* < 0.05.

To test whether the brain-infiltrating SARS-CoV-2 S1 protein is involved in the neurological problems observed in COVID-19 patients, we directly introduced S1 proteins into the dorsal hippocampus, a brain sub-region critical for cognition and emotion^10^, of mice and subjected the mice to a series of behavioral tests to measure cognitive and affective brain functions (Fig. 1a). In novel object recognition and novel location tests, the S1 protein-injected mice exhibited reduced discrimination capacity compared to the vehicle-injected control mice (Fig. 1b). In contrast, locomotive function, which was measured by the total distance traveled during the behavioral session, was not significantly altered. These results indicate that the S1 protein of SARS-CoV-2 in the hippocampus affected mouse cognitive brain function. In the elevated plus maze test, the S1 protein-injected mice spent less time in the center and explored more in the closed arm compared to the control group (Fig. 1c). In addition, in the open field test, the S1 protein-injected group spent less time in the center of the chamber and spent more in the periphery compared to control mice, manifesting anxiety-like behavior (Fig. 1d). Taken together, the hippocampal injection of SARS-CoV-2 S1 protein leads to cognitive deficits and anxiety-like behavior in mice.

### SARS-CoV-2 spike protein induces hippocampal neuronal death and glia activation

**Figure 2:**
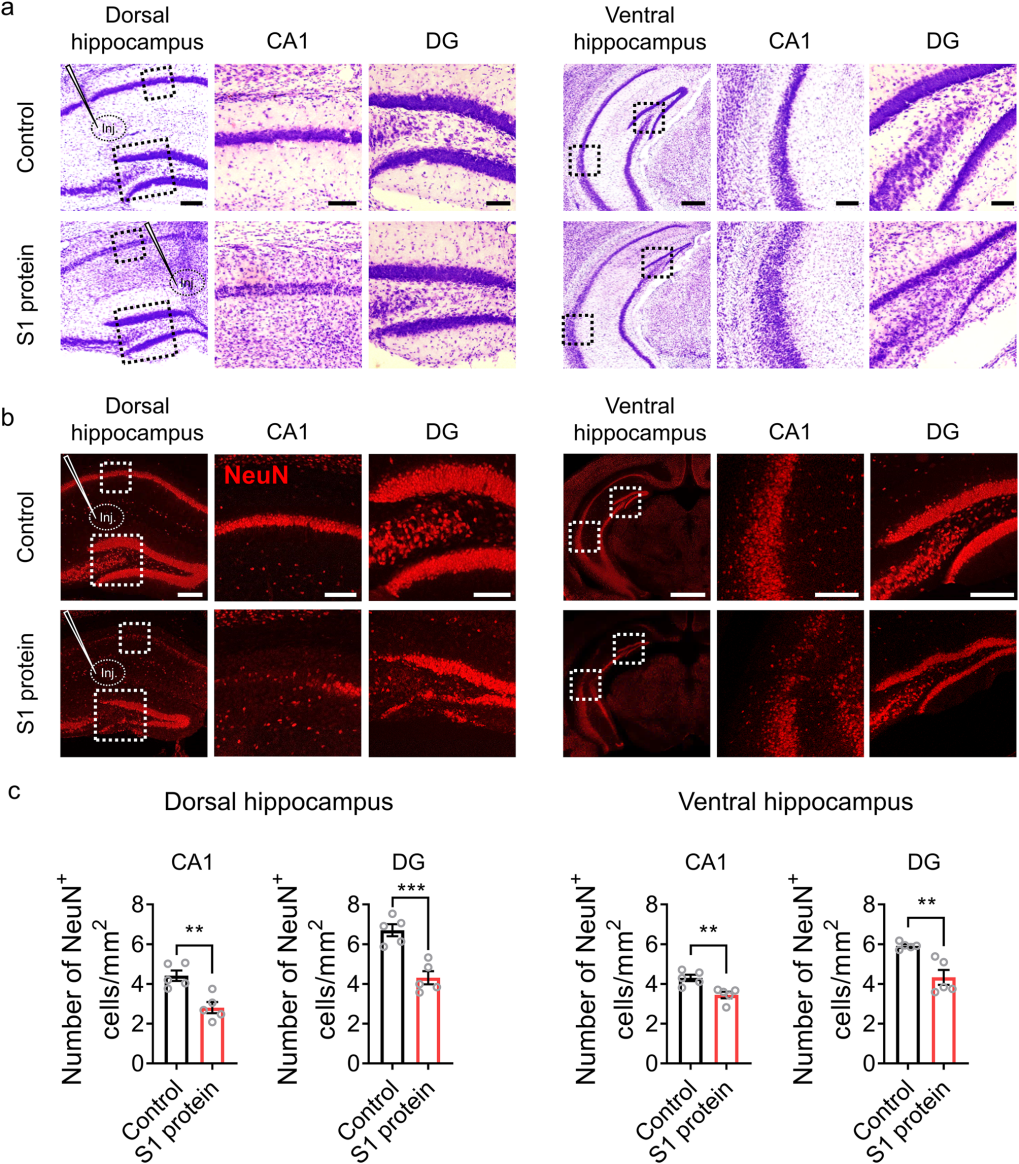
SARS-CoV-2 S1 protein induces hippocampal neuronal death in CA1 and DG areas. (**a-b**). Hippocampal neurons in mice injected with S1 protein (n = 5) or vehicle (Control, n = 5) were visualized by cresyl violet staining (purple, scale bars = 200, 50, and 200 µm for left, middle, and right of dorsal area; 500 µm for left and 125 µm for middle and right of ventral area) and immunostaining with NeuN antibody (red, scale bars = 200, 50, and 100 µm for left, middle, and right of dorsal area; 500 µm for left and 125 µm for middle and right of ventral area). Hippocampal slices were prepared 14 days post-injection. (**c**) NeuN-positive cells in the CA1 and DG regions of the dorsal and ventral hippocampus were manually counted using ImageJ software (Wayne Rasband, National Institutes of Health, USA) in a blind manner. Data are presented as the mean ± s.e.m. Statistical results are for unpaired *t*-tests. **** *p* < 0.0001, ** *p* < 0.01, * *p* < 0.05.

We then evaluated whether the SARS-CoV-2 spike protein can lead hippocampal neuronal cell death. In cresyl violet staining, a markedly reduced neuronal cell density was observed in the CA1 and DG areas of the dorsal and ventral hippocampus in the S1 protein-injected mice (Fig. 2a). The effects of SARS-CoV-2 S1 protein on hippocampal neuronal death was further confirmed by immunohistochemistry (Fig. 2b). Quantifying NeuN-positive neurons demonstrated that S1 protein injection reduced dorsal hippocampal neurons by 35% in both the CA1 and the DG regions and reduced ventral hippocampal neurons by 20% (Fig. 2c). These results show that the SARS-CoV-2 S1 protein induced hippocampal neuronal cell death and implies that the brain-penetrating SARS-CoV-2 spike protein may exert neurotoxic effects in COVID-19 patients.

**Figure 3:**
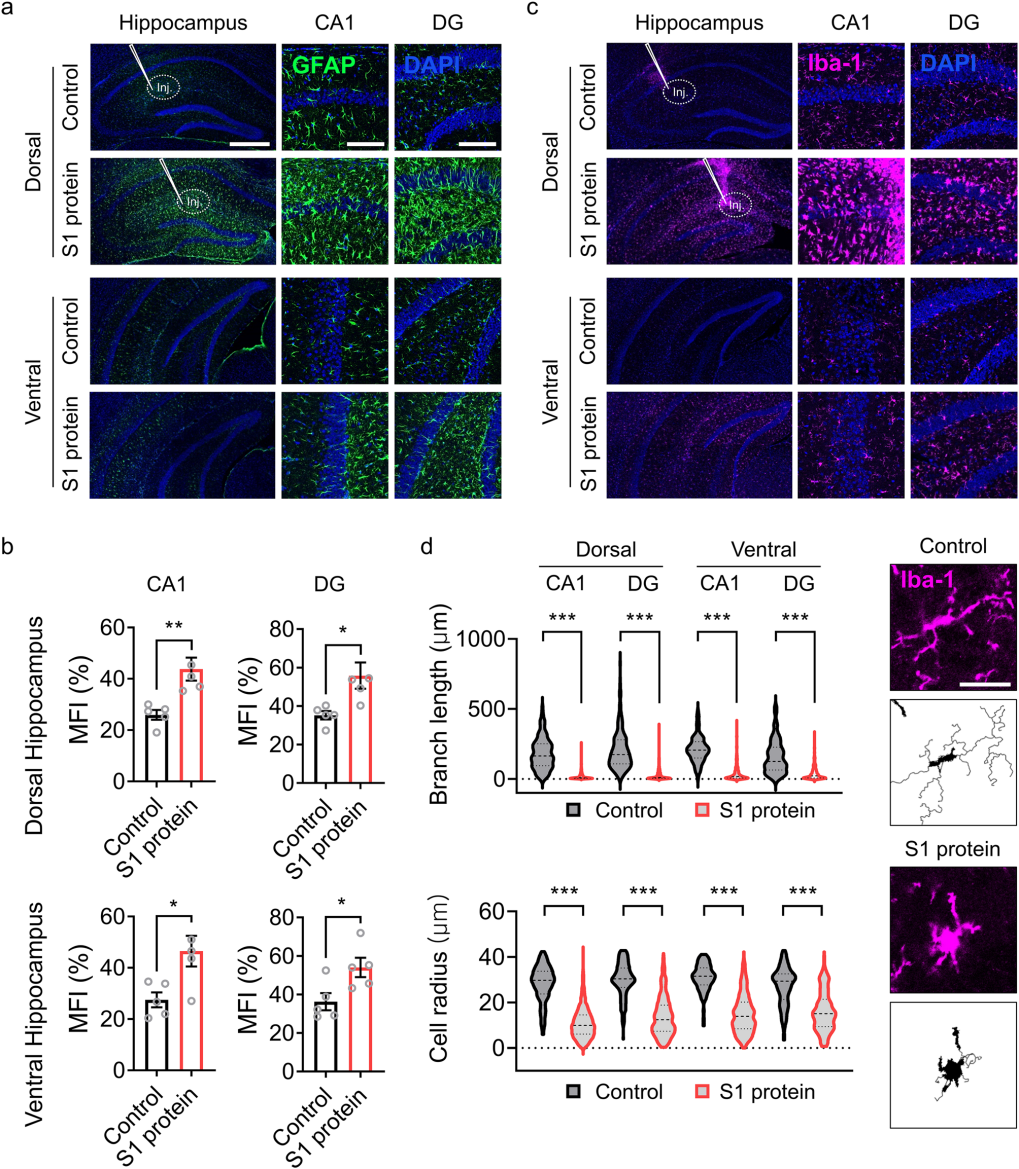
S1 protein induces astrocyte and microglia activation in the hippocampus. (**a, c**) Representative images of immunohistochemistry with GFAP and Iba-1 antibodies show activation of astrocytes (GFAP^+^, green) and microglia (Iba-1^+^, purple) after stereotaxic S1 protein administration (n = 5, scale bar = 500 for left and 250 µm for middle and right). (**b**) Mean fluorescence intensity (MFI) of GFAP^+^ signal measured in CA1 and DG regions of dorsal hippocampus (upper) and ventral hippocampus (bottom). (**d**) Microglia activation was characterized by morphological parameters such as total branch length (upper left) and radius of cell area (bottom left). Representative pictures of microglia in the saline (Control)- or S1 protein-injected mice (right, scale bar = 25 µm). Data are presented as the `mean ± s.e.m. Statistical results are for unpaired *t*-tests. *** *p* < 0.001.

A recent clinical study described an increased glial cell number as well as decreased neuronal cell density in the brains of COVID-19 patients^11^. Therefore, we assessed the effects of SARS-CoV-2 S1 protein on glial cell activation by immunohistochemistry using GFAP and Iba-1 antibodies, which are specific markers for astrocytes and microglia, respectively. Following S1 protein administration in the dorsal hippocampus, GFAP-immunoreactive astrocytes were notably increased in the dorsal hippocampus (Fig. 3a). The fluorescence intensity of GFAP signals increased by 59 and 63% in both the dorsal and ventral hippocampus of CA1 and DG regions (Fig. 3b). Similarly, a dramatic increase in Iba-1 immunoreactivity was detected in the dorsal hippocampus of S1 protein-injected mice (Fig. 3c). Morphologically, the microglia in the S1 protein-injected mice manifested more circular cell bodies and shorter branch lengths than the microglia in the control mice; these are key morphological features of reactive microglia^12^ (Fig. 3d). Taken together, our data demonstrate that the administration of SARS-CoV-2 S1 protein to the hippocampus induces glial cell activation as well as neuronal cell death.

### IL-1β released from the activated glia induce hippocampal neuronal cell death

**Figure 4:**
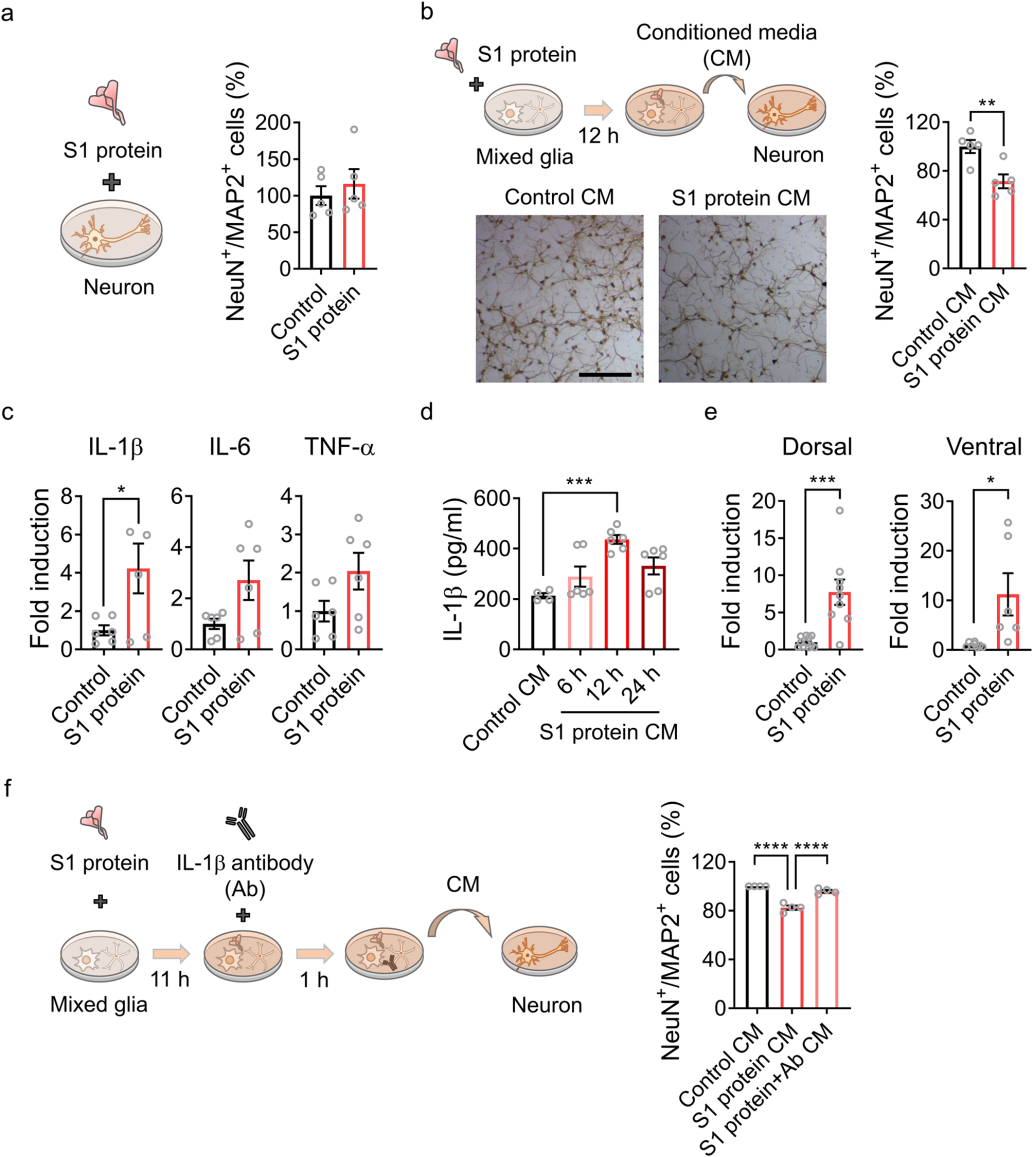
S1 protein induces non-cell autonomous hippocampal cell death via IL-1β induction in glial cells. (**a**) Primary hippocampal neurons were stimulated with S1 protein (5 µg/ml) for 12 h, and NeuN^+^ and MAP2^+^ cells were manually and blindly counted. The relative cell numbers in S1 protein-treated samples compared to the control samples are shown (right). (**b**) A schematic diagram (upper) showing primary hippocampal neuron stimulation by S1 protein-activated glia-conditioned media (CM). After 12 h, neuronal cells were counted, and relative cell numbers in the CM-treated samples are presented (right). Representative images of hippocampal neurons with or without CM treatment for 12 h (bottom). (**c**) Primary glia were stimulated with or without S1 protein for 12 h. Total RNA was prepared, and transcripts of proinflammatory cytokines were measured by real-time RT-PCR analysis. The gene expression levels were normalized against GAPDH in each target and are presented as fold induction. (**d**) IL-1β protein levels in the CM of primary glia with or without S1 treatment were measured by ELISA. (**e**) IL-1β expression in the hippocampus in vivo 6 h after administration of S1 protein. IL-1β transcript levels in the dorsal and ventral hippocampus were separately examined. (**f**) An experimental scheme testing the neurotoxicity of glial cell-derived IL-1β in CM using IL-1β-neutralizing antibody. Pretreatment with IL-1β antibody for 1 h rescues hippocampal neuronal death by CM. Data are presented as the mean ± s.e.m. Statistical results are from unpaired *t*-tests. **** *p* < 0.0001, *** *p* < 0.001, * *p* < 0.05.

Next, the neurotoxic mechanism of the SARS-CoV-2 spike protein was investigated by treating hippocampal neurons with the S1 protein in vitro. Incubation with S1 protein (5 µg/ml) for 12 h did not affect the survival rate of primary cultured hippocampal neurons (Fig. 4a), suggesting the non-cell autonomous neurotoxicity of the SARS-CoV-2 spike protein. We then incubated primary hippocampal neurons in the conditioned medium from mixed glia that were stimulated with S1 protein to test whether S1 protein-induced neuronal cell death was mediated by activated glia. After 12 h of incubation in the conditioned medium, the hippocampal neuronal cell number was significantly decreased (Fig. 4b), indicating that certain factor(s) derived from the S1 protein-activated glial cells induced neuronal cell death. Studies have shown that glial cells produce neurotoxic molecules such as pro-inflammatory cytokines under pathogenic conditions^13^. The pro-inflammatory cytokine expression in the primary glia upon SARS-CoV-2 S1 protein stimulation was tested to identify the putative molecules that are derived from the activated glia and mediate hippocampal neuronal death. The transcription of IL-1β, a potentially neurotoxic inflammatory cytokine^14^, was induced in primary glial cells by S1 protein stimulation (Fig. 4c). In addition, the release of IL-1β into conditioned media from the activated primary glial cells was also confirmed (Fig. 4d). IL-1β expression was also up-regulated more than sevenfold in dorsal as well as ventral hippocampi by S1 protein injection in vivo (Fig. 4e). These data suggest that the IL-1β released by spike protein-activated glia may induce hippocampal neuronal cell death. This was tested by incubating the conditioned media with IL-1β-neutralizing antibody before transfer to the primary hippocampal neurons. Depleting IL-1β from the conditioned media almost completely rescued the S1 protein-mediated hippocampal neuronal cell death (Fig. 4f). Taken together, our data indicate that the SARS-CoV-2 spike protein causes non-cell autonomous hippocampal neuronal cell death by inducing IL-1β expression from glial cells.

## Discussion

Many clinical reports of COVID-19 survivors suffering from various neurological and psychiatric symptoms such as cognitive decline and mood disorder undoubtedly show that SARS-CoV-2 affects the human CNS. The initial assumption of direct SARS-CoV-2 infection in the brain was not supported by following studies; thus, the cellular and molecular mechanisms are still elusive. The recent discovery of the blood–brain barrier-penetrating SARS-CoV-2 spike protein^9^ offers a new possibility that the SARS-CoV-2-derived spike protein causes neurological or psychiatric symptoms observed in the COVID-19 survivors, which we tested in this study. We demonstrated that S1 protein administration into mice hippocampi led to cognitive decline and anxiety-like behavior. These data suggest that several neurological and psychiatric symptoms observed in COVID-19 patients can be mediated by the brain-penetrating S1 protein in the absence of direct CNS infection of SARS-CoV-2.

It is well known that the hippocampus is important for cognition and emotions^15,16^. Specifically, the dorsal hippocampus is involved in cognition as well as learning and memory^17,18^ and the ventral hippocampus in affective brain functions such as anxiety^19^. Therefore, it is easily conceivable that hippocampal neuronal cell death in these areas due to S1 protein administration may lead to the cognitive deficits and anxiety-like behavior observed in our study. Notably, angiotensin-converting enzyme 2 (ACE2) and neuropilin-1, two receptors for S1 protein, are relatively highly expressed in the hippocampus as compared to other brain regions^20,21^. This may increase the susceptibility of the hippocampus to the S1 protein effects and might explain why hippocampus-dependent neurological and psychological symptoms such as cognitive deficit or anxiety and depression often manifest in COVID-19 patients.

In our effort to elucidate the mechanisms, we found that S1 protein induced non-cell autonomous hippocampal neuronal cell death. The S1 protein exerted hippocampal neurotoxicity via activating glial cells. More specifically, we found that IL-1β expressed by the S1 protein-activated glia contributes to hippocampal neuronal cell death. The inflammatory activation of glia and subsequent bystander neuronal cell death have been often observed in other neurotropic virus infections such as HIV and Japanese encephalitis virus infection, in which glia-expressed IL-1β was implicated^22–24^. Previously, the S1 protein of coronavirus was shown to induce inflammatory cytokines by activating NF-$\kappa$B signaling in microglia^25^, and ACE2 and CD147, receptors that interact with S1, are expressed on glial cells^20,26^. Taking into account these prior studies, our data suggests that the brain-penetrating S1 protein of SARS-CoV-2 can activate hippocampal glial cells to express IL-1β and thereby induce bystander hippocampal neuronal cell death.

In summary, we present evidence that the S1 protein of SARS-CoV-2 introduced into the hippocampus can induce non-cell autonomous hippocampal neuronal death resulting in cognitive deficit and anxiety-like behavior. This novel mechanism may open a new avenue for the treatment of neurological and psychological symptoms of COVID-19 survivors in the future.

## Materials and methods

### Animals

Eight-to-ten weeks old male C57BL/6 mice, weighing 21–25 g, were obtained from Daehan Biolink Co. Ltd. (Chungbuk, Korea) and randomly divided into animal cages. Animals were housed and maintained in a controlled environment at 22–24 °C and 55% humidity with 12 h light/dark cycles and fed regular rodent chow and tap water ad libitum. All animal care was guided by the Seoul National University Institutional Animal Care and Use Committee (SNU IACUC) to minimize pain and distress during experimental interventions and all animal experimental procedures were performed following the Animal Research: Reporting In Vivo Experiments (ARRIVE) guidelines.

### Stereotaxic spike protein delivery to mice hippocampi

Following a previous study, hippocampal administration of S1 protein was performed^27^. Eight-week-old mice were used for the spike protein injections. S1 protein was purchased from Acrobiosystems (Cat #S1N-C52H4, Newark, DE, USA), and used after polymixin B (Cat #P1004, Sigma-Aldrich, St. Louis, MO, USA) treatment (30 µg/ml). The animals were anesthetized with isoflurane, and the injection paths were drilled into their skulls. Then, 5 µg of S1 protein (1 µg/µl) were bilaterally injected into each hippocampal region using a Hamilton syringe (Cat #80330, Hamilton Company, Reno, NV, USA) attached to a syringe pump (Cat #53311, Stoelting Co., Wood Dale, IL, USA) at a constant volume of 0.5 µl/min. The injection coordinates of the hippocampus were 1.5 (ML), − 2.06 (AP), and − 2.0 (DV) from the bregma.

### Novel object recognition and location recognition test

The novel object recognition and location recognition tests were performed using previously described methods^28,29^ with minor modifications. First, each mouse was placed on one side of the open field box and allowed to freely explore for 10 min. After one day, two identical objects were presented to the mouse for 10 min. Then, one object was replaced with a novel object, and the mouse was allowed to explore for 5 min. Subsequently, one object was replaced in a novel location, and the mouse was allowed to explore for 5 min. The chamber and objects were cleaned with 70% ethanol between trials to remove olfactory stimuli. Testing sessions were video monitored, and object exploration times were scored by a blinded experimenter. Results are expressed as the discrimination ratio of time spent with the novel object or novel location to the total exploration time. The discrimination index of the novel object or location was calculated by the following formula: (discrimination time of novel object or location/total discrimination time of novel and familiar object or location)*100. The exploration time were measured automatically by SMART 3.0 software (Panlab, Barcelona, Spain).

### Elevated plus maze (EPM)

The EPM was used to examine anxiety-like behavior. The behavioral apparatus consisted of two open arms and two closed arms elevated 50 cm above the floor. Mice were placed individually in the center of the maze, facing an open arm, and allowed to freely explore for 5 min. The maze was cleaned with 70% ethanol after each test to prevent influence from the previously tested mouse. The times spent in the open and closed arms were measured automatically by SMART 3.0 software.

### Open field test (OFT)

The OFT was performed as previously described^30^. Each mouse was placed in the center of an open arena and allowed to freely explore the arena for 5 min. The times spent in the center and the periphery zone were automatically measured by SMART 3.0 software.

### Cresyl violet staining

Cresyl violet (Cat #C5042, Sigma-Aldrich) staining was performed as previously described with minor modification^31^. Briefly, brain sections were rehydrated with serially diluted ethanol (from 100 to 70%) for 2 min and with xylene for 5 min. Rehydrated tissues were incubated with 0.1% cresyl violet solution for 10 min. After the solution was washed away with distilled water, tissues were dehydrated with diluted ethanol (from 70 to 100%) for up to 1 min for each. The samples were rinsed in xylene and mounted on slides.

### Immunohistochemistry

Immunostaining was carried out using previously established protocols^32^. Briefly, the brain sections were incubated in a blocking solution with 5% normal goat serum (Cat #005–000-121, Jackson ImmunoResearch, West Grove, PA, USA), 2% bovine serum albumin (Cat #A6059, Sigma-Aldrich) and 0.1% Triton X-100 (Cat #0694, VWR, Avantor, Radnor, PA, USA) for 1 h at room temperature (RT). Tissue samples were then incubated overnight at RT with primary antibody for rabbit-anti-Iba1 (Cat #019–19741, 1:1000; Wako, Osaka, Japan) and mouse-anti-GFAP (Cat #MAB360, 1:2000; Millipore, Darmstadt, Germany) or NeuN (Cat #MAB377, 1:1000; Millipore). After being rinsed in 0.1 M PBS, the samples were incubated for 1 h at RT with a mixture of Cy3- or FITC-conjugated secondary antibodies (Cat #111–165-003 and #115–095-020, 1:200; Jackson ImmunoResearch) and mounted with VectaShield medium (Cat #H-1500 Vector Labs, Burlingame, CA, USA). Fluorescence images were obtained using a confocal microscope (LSM800; Carl Zeiss, Oberkochen, Germany) and fluorescence intensity were automatically analyzed by Zen software (Carl Zeiss, Oberkochen, Germany).

### Microglia morphological analysis

Morphological analysis of microglia was performed using a method of MATLAB (version R2021a, The MathWorks Inc., Natick, MA, USA) as described previously^33^. Briefly, by tracking microglial soma and processes, intensity quantiles across the image were identified, and we then quantified the resultant image. To normalize fluorescence intensities between tissue samples, the quantile level of the background and the soma intensity were automatically modified. Minimum object recognition (i.e., soma) was set to 200–300 pixels. At 20 × magnification, the total area of view (320 µm^2^) of the region of interest (hippocampal CA1 and DG) was evaluated.

### Primary cell culture

E17-18 embryos and one-day-old C57BL/6 pups were used to culture primary glia and hippocampal neurons using a procedure described previously^34,35^. In primary mixed glia culture, after removing the meninges from the cerebral hemisphere, tissue was dissociated into a single-cell suspension through gentle repetitive pipetting. Cells were then cultured in Dulbecco's Modified Eagle's medium (Cat #LM001-01, Welgene, Gyeongbuk, Korea) supplemented with 10% fetal bovine serum (Cat #16000–044, Gibco Laboratories, Grand Island, NY, USA), l-glutamine (Cat #25030081, Gibco Laboratories), non-essential amino acids (Cat #11140050, Gibco Laboratories), and antibiotic/antimycotic (Cat #15240062, Gibco Laboratories) in 75-cm^2^ flasks (Cat #156499, Nunc, Rochester, NY, USA) at 37 °C in a 5% CO_2_ incubator, and the medium was changed every five days. For primary neuron culture, mice hippocampi were dissected and incubated in 2.5% trypsin buffer (Cat #15090–046, ThermoFisher Scientific, Waltham, MA, USA) for 15 min. After being transferred to fresh culture media, hippocampi tissue was dissociated into a single-cell suspension through gentle repetitive pipetting. After counting, cells were cultured in neurobasal medium supplemented with l-glutamine, penicillin streptomycin (Cat #15140–122, Gibco Laboratories), and B-27 supplements (Cat #17504–044, Gibco Laboratories) for 10 days before use for experiments.

### 3,3′-Diaminobenzidine (DAB) staining

DAB staining was conducted using published protocols with minor modification^36^. Briefly, hippocampal neurons were fixed with 4% paraformaldehyde and then incubated with antibodies for neuronal cell markers (NeuN, 1:1000, and MAP2, Cat #AB5622, 1:1000, Millipore) after blocking. After incubation with biotinylated anti-mouse-IgG antibody (Cat #B7264, Vector Labs, Burlingame, CA, USA) and streptavidin–horseradish peroxidase (HRP) conjugates (Cat #554066 ThermoFisher Scientific), the cells were treated with DAB (Cat #D12384, Sigma-Aldrich) for 30 min of reaction time.

### Real-time RT-PCR

Total RNA was isolated from hippocampal tissue and cells using TRIzol reagent (Cat #15596018, Invitrogen, Carlsbad, CA, USA), and 1–2 µg of total RNA were used for cDNA synthesis. Amplification of each gene was performed using an ABI Prism 7500 sequence detection system (Applied Biosystems, Foster City, CA, USA) and SYBR Green PCR Master Mix (Cat #4309155, Applied Biosystems) as previously described^37^. Following the 2^-ΔΔCt^ method^38^, relative mRNA levels were calculated, and delta-Ct values of each gene mRNA level were normalized to that of GAPDH. All real-time RT-PCR experiments were performed at least three times, and the mean ± s.e.m. values are presented unless otherwise noted. The following PCR primer sequences were used: GAPDH forward, 5′-AGG TCA TCC CAG AGC TGA ACG-3′; GAPDH reverse, 5′-CAC CCT GTT GCT GTA GCC GTA-3′; IL-1β forward, 5′-GTG CTG TCG GAC CCA TAT GA-3′; IL-1β reverse, 5′-TTG TCG TTG CTT GGT TCT CC-3′; IL-6 forward, 5′-CCA CGA TTT CCC AGA GAA CAT-3′; IL-6 reverse, 5′-TCC ATC CAG TTG CCT TCT TGG-3′; TNF-α forward, 5′-AGC AAA CCA CCA AGT GGA GGA-3′; and TNF-α reverse, 5′-GCT GGC ACC ACT AGT TGG TTG T-3′.

### Enzyme‐linked immunosorbent assay (ELISA)

The expression levels of IL-1β protein from mixed glia stimulated with S1 protein (5 µg/ml) for 6, 12, and 24 h were examined with an ELISA kit (Cat #MLB00C, R&D Systems Inc., Minneapolis, MN, USA) following the manufacturer’s protocols. The conditioned media from each time point was assessed in duplicate. Optical density was measured at 450 nm by a SpectraMax ABS Plus microplate reader (Molecular Devices, San Jose, CA, USA).

### Statistical analysis

Statistical analyses were performed and graphs were made using GraphPad Prism 7.0 for Windows software (GraphPad Software Inc., La Jolla, CA, USA). The data were analyzed using an unpaired t-test as appropriate to assess significant differences. All data are expressed and plotted as the mean ± s.e.m. A p-value less than 0.05 was considered statistically significant. No animals or data point were excluded from the analysis. No statistical methods were used to pre-determine sample sizes, but our sample size is similar to those generally employed in the field.

### Ethic declaration

This study was conducted according to the The Institutional Animal Care and Use Committee (IACUC) of Seoul National University (SNU) approved the study protocol (Approval number # SNU-210114-3-2).
